# Evaluation of Buspirone on Streptozotocin Induced Type 1 Diabetes and Its Associated Complications

**DOI:** 10.1155/2014/948427

**Published:** 2014-01-20

**Authors:** Suchi Raghunathan, Pratik Tank, Shraddha Bhadada, Bhoomika Patel

**Affiliations:** Institute of Pharmacy, Nirma University, Sarkhej-Gandhinagar Highway, Ahmedabad, Gujarat 382 481, India

## Abstract

We have evaluated the effect of buspirone (1.5 mg/kg/day, p.o.) type 1 diabetes induced cardiovascular complications induced by streptozotocin (STZ, 45 mg/kg, i.v.) in Wistar rats. Various biochemical, cardiovascular, and hemodynamic parameters were measured at the end of 8 weeks of treatment. STZ produced significant hyperglycaemia, hypoinsulinemia, and dyslipidemia, which was prevented by buspirone treatment. STZ produced increase in serum creatinine, urea, lactate dehydrogenase, creatinine kinase, and C-reactive protein levels and treatment with buspirone produced reduction in these levels. STZ produced increase in cardiac and LV hypertrophy index, LV/RV ratio, and LV collagen, which were decreased by buspirone treatment. Buspirone also prevented STZ induced hemodynamic alterations and oxidative stress. These results were further supported by histopathological studies in which buspirone showed marked reduction in fibrosis and cardiac fiber disarray. In conclusion, our data suggests that buspirone is beneficial as an antidiabetic agent in type 1 diabetes mellitus and also prevents its cardiac complications.

## 1. Introduction

Type 1 diabetes is characterized by deficient insulin production and requires daily administration of insulin. According to World Health Organization (WHO), death due to diabetes will double between 2005 and 2030 [1]. Worldwide, 346 million people are suffering from diabetes and more than 80% of diabetes deaths occur in low- and middle-income countries. The new FDA guideline recommends sponsors to demonstrate that the newer antidiabetic drug therapy would not result in an unacceptable increase in CVS risk [2]. Thus, there is an imperative need for newer antidiabetic drugs which would be cardioprotective or less cardiotoxic.

It has been reported that 5-hydroxytryptamine (5-HT) is involved in glycemic control. Antagonism of 5-HT_1A_ receptor by antipsychotics like, clozapine, olanzapine, or risperidone results in impairment of glucose homeostasis (hyperglycemia). Buspirone is partial agonist at 5-HT_1A_ receptor and also has affinity for brain dopamine D_2_ receptors [3]. Buspirone through its major metabolite 1-(2-pyrimidinyl) piperazine (1-PP) also shows *α*_2_ antagonist activity [4]. Various studies have been carried out to evaluate the effect of buspirone on glycemic status. Sugimoto et al. reported that buspirone (10 mg/kg) reduced immobilization stress, induced hyperglycemia, and raised serum insulin levels of nonstressed and stressed mice [5]. They also reported that 1-PP, the major metabolite of buspirone inhibited stress, induced hyperglycemia and increased serum insulin levels of stressed mice [5]. In a clinical study, Ojha et al. [6] reported that buspirone (10 mg/kg, p.o.) produced a significant decrease in blood glucose level postprandial without altering insulin level and had no significant effect on fasting blood glucose and plasma insulin level in health human male volunteers. Recent reports have revealed that buspirone (20 mg/kg, i.p.) significantly reduced serum glucose level in diabetic adult Wistar rats but did not significantly affect serum insulin and C-peptide level compared to diabetic control values [7]. Buspirone has been associated with few side effects like tachycardia, palpitation, nervousness, gastrointestinal distress, paresthesias, and a dose-dependent pupillary constriction.

Many reports of buspirone on glycemic control and stress induced hyperglycemia are available. However, till date, there is no data available on effect of buspirone on glycemic control in diabetes and its related complications. Hence, the aim of present investigation was to evaluate the role of buspirone in reducing the complications of type 1 diabetes induced by streptozotocin in a rat model.

## 2. Material and Methods

The protocol of the experiment was approved by our institutional animal ethical committee as per the guidance of the Committee for the Purpose of Control and Supervision of Experiments on Animals (CPCSEA), Ministry of Social Justice and Empowerment, Government of India (IPS/PCOL/MPH10-11/005 dated on 7th of August, 2010).

### 2.1. Induction of Type 1 Diabetes

Healthy adult Wistar rats of either sex weighing 180–220 g of 6–8 weeks of age were chosen for the study and maintained under well-controlled conditions of temperature (22 ± 2°C), humidity (55 ± 5%), and 12 h/12 h light-dark cycle. Standard laboratory rat chow and UV-filtered water were provided *ad libitum*. The rats were made diabetic by intravenous (i.v.) injection with 45 mg/kg streptozotocin (STZ, Sigma Ltd., USA) dissolved in citrate buffer (0.1 M, pH-4.5). Two days after the injection of STZ, animals were checked for urine glucose levels with the help of available diagnostic kit-Diastix (Bayer Healthcare, India). Rats displaying urine glucose levels > 250 mg/dL were considered as diabetic. The rats were then randomly divided into four groups: control (CON), control treated with buspirone (COB), diabetic control (DIC), and diabetics treated with buspirone (DIB). Buspirone was dissolved in distilled water and was administered orally (p.o.) at a dose of 1.5 mg/kg/day for eight weeks in control treated and disease treated groups. All animals were monitored regularly for changes in body weight and mortality throughout the course of study.

### 2.2. Blood Sample and Serum Analysis

At the end of eight weeks, rats were fasted for 16 h after which distilled water (normal control and diabetic control) and buspirone (1.5 mg/kg to the control treated and diabetic treated) were then orally administered to groups of 6 rats each. Thirty minutes later, glucose (3 g/kg) was orally administered to each rat and blood samples were collected from the tail vein at −30 (just before the buspirone administration), 0 (just before the oral administration of glucose), 30, 60, 120, and 180 min after glucose load for the oral glucose tolerance test (OGTT). Blood samples were collected at the end of eight weeks from the retroorbital plexuses of each rat under light ether anesthesia. Serum was separated and was analyzed for glucose, OGTT, glycosylated haemoglobin, triglycerides, total cholesterol, very low density lipoprotein-cholesterol (VLDL-C), low density lipoprotein-cholesterol (LDL-C), high density lipoprotein-cholesterol (HDL-C), C-reactive protein (CRP), lactate dehydrogenase (LDH), creatinine kinase-MB (CK-MB), creatinine, and blood urea nitrogen spectrophotometrically (Shimadzu UV-1601, Japan) using available biochemical diagnostic kits (Lab-Care Diagnostics Pvt. Ltd., India). Serum insulin was estimated by radioimmunoassay technique using diagnostic kits (Anand Brothers and AB Diachem Systems Pvt. Ltd., New Delhi, India) in multiple well gamma counter.

### 2.3. Measurement of Invasive Hemodynamics

At the end of eight weeks, various hemodynamic parameters were recorded following the procedure of carotid artery cannulation. The animals were briefly anaesthetized by Ketamine (20 mg/kg, i.p.) + xylazine (10 mg/kg, i.m.). The body temperature was maintained at 37 ± 1°C during the experiment. The carotid artery behind the trachea was exposed and cannulated for the measurement of hemodynamic parameters using a transducer (BP 100) and Labscribe Systems (IWORX, New Hampshire, USA). The hemodynamic parameters observed were mean arterial blood pressure (MABP), heart rate, rate of pressure development (dp/dtmax), and rate of pressure decay (dp/dtmin). All the data were analyzed using Labscribe software (Version 118).

### 2.4. Hypertrophy Assessment

After measurement of hemodynamic parameters, animals were sacrificed, hearts were excised, extraneous tissues were removed, and wet weight of the heart, left ventricle, and right ventricle along with femur length was noted down to calculate index of cardiac hypertrophy (CHI) and left ventricular (LV) hypertrophy index (LVHI). Further, left ventricular weight to right ventricular weight ratio (LVW/RVW) and heat weight to body weight ratio (HW/BW) were also estimated. Left ventricular (LV) wall thickness was measured using screw gauge micrometer.

### 2.5. Statistical Analysis

Results are expressed as mean ± SEM. Statistical differences between groups were applied using SPSS software version 17.0 (USA). Data were considered to be statistically significant at *P* value < 0.05.

## 3. Results

### 3.1. General Parameters of Experimental Rats

Injection of STZ (45 mg/kg) into rats produced cardinal signs of type 1 diabetes in all the animals. STZ diabetic rats showed a loss of body weight, polyphagia, and polydypsia. Chronic treatment with buspirone produced a significant (*P* < 0.05) increase in body weight and significant decrease in food and water intake in STZ diabetic treated rats as compared to STZ diabetic control rats (Table 1).

### 3.2. Biochemical Parameters

**Figure 1:**
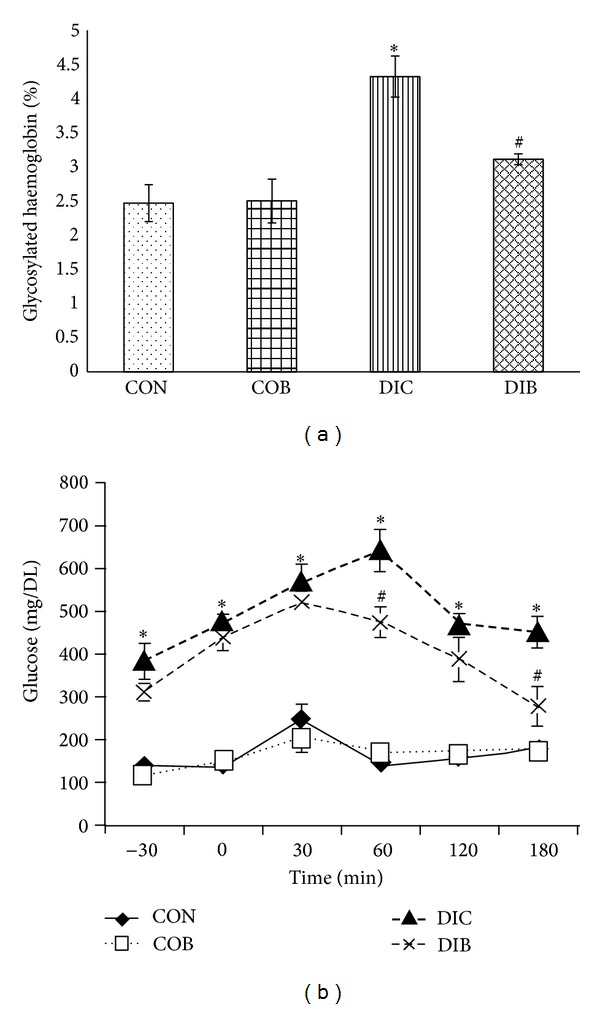
Effect of buspirone on change in (a) glycosylated hemoglobin levels and (b) oral glucose tolerance test (OGTT). *Significantly different from normal control (*P* < 0.05). ^#^Significantly different from diabetic control (*P* < 0.05). Each bar represents mean ± SEM of 6 experiments. CON: normal control, COB: control treated with buspirone, DIC: diabetic control, and DIB: diabetic treated with buspirone.

**Figure 2:**
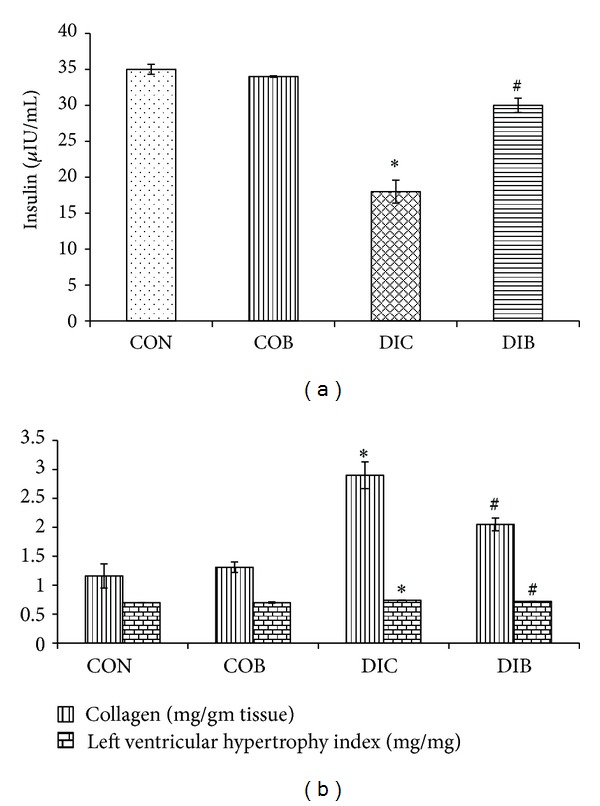
Effect of buspirone on (a) insulin levels and (b) collagen and LV hypertrophy index. *Significantly different from normal control (*P* < 0.05). ^#^Significantly different from diabetic control (*P* < 0.05). Each bar represents mean ± SEM of 6 experiments. CON: normal control, COB: control treated with buspirone, DIC: diabetic control, and DIB: diabetic treated with buspirone.

STZ diabetic rats were found to exhibit significant hyperglycemia, hypoinsulinemia, and a significant increase in percent glycosylated haemoglobin levels as compared to control rats. Treatment with buspirone produced a significant (*P* < 0.05) decrease in the elevated serum glucose levels (Table 1), percent glycosylated haemoglobin levels (Figure 1(a)), and decreased glucose levels after 2 hours of glucose load supported by oral glucose tolerance test (Figure 1(b)) accompanied with significant increase in insulin levels in STZ diabetic treated rats as compared to STZ diabetic control rats (Figure 2(a)).

Additionally, a significant (*P* < 0.05) increase in serum creatinine and urea levels was observed in STZ diabetic rats as compared to control rats. Chronic treatment with buspirone resulted in a significant (*P* < 0.05) decrease of serum creatinine and urea levels in STZ diabetic treated rats as compared to STZ diabetic control rats (Table 1).

There was a significant (*P* < 0.05) increase in triglycerides, total cholesterol, VLDL-C, and LDL-C levels and significant decrease in HDL-C levels in STZ diabetic rats as compared to control rats. Treatment with buspirone significantly (*P* < 0.05) reduced the elevated triglyceride, total cholesterol, VLDL-C, and LDL-C levels in STZ diabetic treated rats as compared to STZ diabetic control rats and significantly increased HDL-C levels (Table 2).

### 3.3. Serum Cardiac Biomarkers

STZ produced a significant (*P* < 0.05) increase in serum LDH and CK-MB levels as compared to control rats. Chronic treatment with buspirone produced a significant (*P* < 0.05) decrease in LDH and CK-MB levels in STZ diabetic treated rats as compared to STZ diabetic control rats (Table 3).

In addition, STZ produced a significant (*P* < 0.05) increase in serum CRP levels as compared to control rats. Chronic treatment with buspirone produced a significant (*P* < 0.05) decrease in CRP levels in STZ diabetic treated rats as compared to STZ diabetic control rats (Table 3).

### 3.4. Hypertrophic Parameters

Cardiac hypertrophic index (CHI) was significantly (*P* < 0.05) higher in STZ diabetic rats as compared to controls. Chronic treatment with buspirone significantly (*P* < 0.05) reduced the elevated CHI (Table 3) in STZ diabetic treated rats as compared to STZ diabetic control rats.

Further, LV hypertrophic index (LVHI) and left ventricular collagen levels were also found to be significantly (*P* < 0.05) higher in STZ diabetic rats as compared to control rats. Chronic treatment with buspirone significantly (*P* < 0.05) reduced the elevated LVHI and LV collagen levels in STZ diabetic treated rats as compared to STZ diabetic control rats (Figure 2(b)).

**Figure 3:**
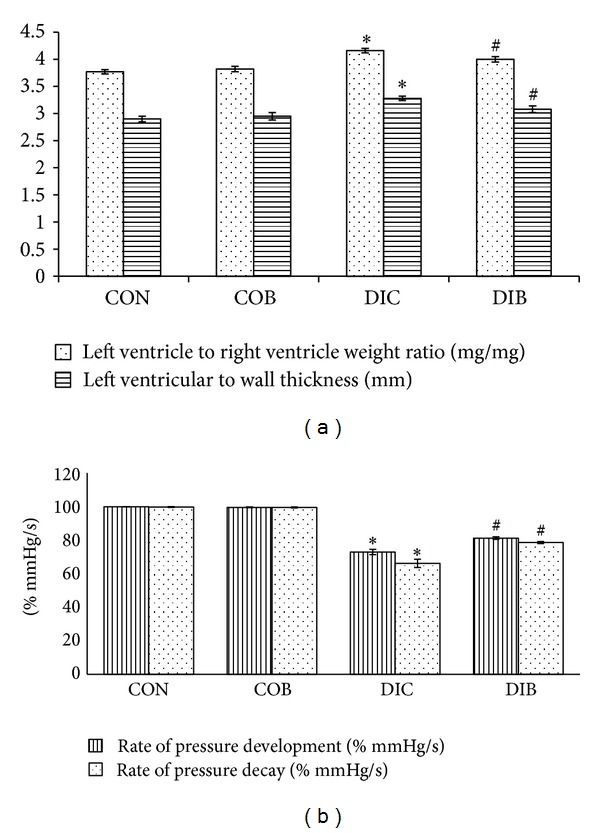
Effect of buspirone on (a) LVW/RVW ratio and LV wall thickness and (b) rate of pressure development and rate of pressure decay. *Significantly different from normal control (*P* < 0.05). ^#^Significantly different from diabetic control (*P* < 0.05). Each bar represents mean ± SEM of 6 experiments. CON: normal control, COB: control treated with buspirone, DIC: diabetic control, and DIB: diabetic treated with buspirone.

Additionally, LVW/RVW ratio and LV wall thickness were also significantly (*P* < 0.05) high in diabetic control rats. Chronic treatment with buspirone significantly (*P* < 0.05) reduced the elevated LVW/RVW ratio and LV wall thickness in STZ diabetic treated rats as compared to STZ diabetic control rats (Figure 3(a)).

### 3.5. Hemodynamic Parameters

STZ diabetic control rats showed a significantly (*P* < 0.05) higher blood pressure and significantly (*P* < 0.05) lower heart rate in diabetic control animals as compared to control animals. Chronic treatment with buspirone significantly (*P* < 0.05) decreased blood pressure and significantly (*P* < 0.05) increased heart rate in STZ diabetic treated rats as compared to STZ diabetic control rats (Table 4).

Further, rate of pressure development and rate of pressure decay were found to be significantly (*P* < 0.05) lower in diabetic control animals as compared to control animals (Figure 3(b)). Chronic treatment with buspirone significantly (*P* < 0.05) increased the rate of pressure development and rate of pressure decay in STZ diabetic treated rats as compared to STZ diabetic control rats (Figure 3(b)).

### 3.6. Oxidative Stress Parameters

Malondialdehyde (MDA) was found to be significantly (*P* < 0.05) higher in STZ-diabetic rats as compared to control rats. Treatment with buspirone produced a significant (*P* < 0.05) reduction in MDA levels (Table 4) in STZ diabetic treated rats as compared to STZ diabetic control rats.

Glutathione (GSH) and superoxide dismutase (SOD) in cardiac tissues were found to be significantly (*P* < 0.05) higher in STZ diabetic control animals as compare to control animals. Chronic treatment with buspirone showed significant (*P* < 0.05) increase in GSH and SOD in cardiac tissues in STZ diabetic treated rats as compared to STZ diabetic control rats (Table 4).

### 3.7. Histopathological Studies

**Figure 4:**
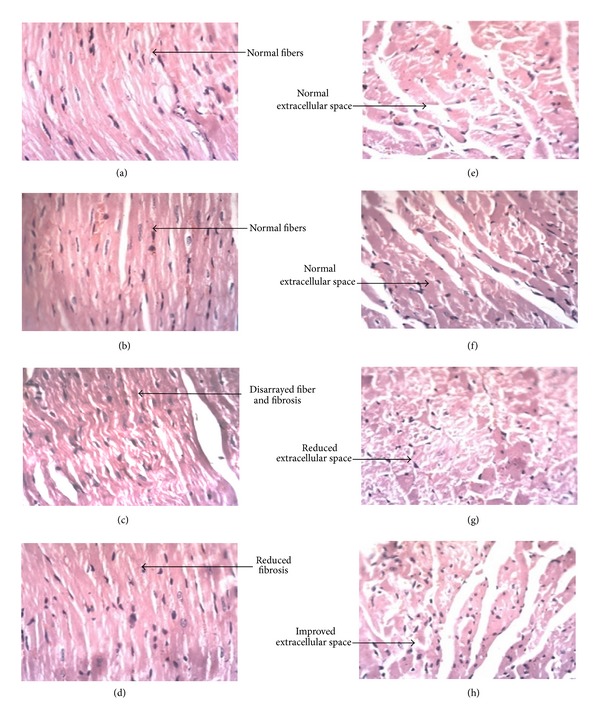
Representative figures of cardiac fibres: (a) normal control; (b) control treated with buspirone; (c) diabetic control; (d) diabetic treated with buspirone and cariomyocytes; (e) normal control; (f) control treated with buspirone; (g) diabetic control; (h) diabetic treated with buspirone.

The histopathological findings of the transverse sections of LV revealed that there was intense fibrosis and cardiac fiber disarray in diabetic control rats as compared to control rats. Treatment with buspirone reduced the intensity of fibrosis and cardiac fiber disarray in STZ diabetic treated rats as compared to STZ diabetic control rats (Figures 4(a), 4(b), 4(c), and 4(d)).

Further, the histopathological findings also revealed that there was reduction in extracellular space and increase in cardiomyocyte diameter in diabetic control rats as compared to control rats. Treatment with buspirone in diabetic treated rats showed a less increase in cardiomyocyte diameter and less reduction in extracellular space STZ diabetic treated rats as compared to STZ diabetic control rats (Figures 4(e), 4(f), 4(g), and 4(h)).

## 4. Discussion

In the present study, streptozotocin (STZ) produced cardinal signs of type 1 diabetes which includes hyperglycaemia, loss of body weight, polyphagia, polydipsia, and polyuria which are consistent with those reported earlier [8, 9]. These cardinal signs of diabetes were significantly reversed by chronic treatment with buspirone.

Streptozotocin is a cytotoxic glucose analogue, which modifies biological molecules, fragments DNA, and destroys the beta cells, causing a state of insulin-dependent diabetes by deprivation of insulin [10]. In the present study, buspirone prevented the hyperglycemia and hypoinsulinemic condition in STZ diabetic rats. It has been reported that pancreatic *β* cells have 5-HT1A receptors and antagonism of these receptors by antipsychotics may decrease pancreatic *β*-cell responsiveness to glucose levels resulting in impairment of glucose homeostasis [11]. Thus, buspirone effect of decrease in glucose levels, being an agonist at 5-HT1A receptor, is justified. Furthermore, the buspirone metabolite, 1-PP, is recognized as an antagonist of *α*-2 receptors with very low affinity for 5-HT1A receptors [12]. The *α*-2 receptors are expressed in *β* cells of islet which inhibit insulin release [13]. Thus, agonism of 5-HT1A receptor mediated by buspirone and *α*-2 antagonist effects of 1-PP might be possible mechanism behind decrease in glucose levels and increase in insulin levels.

Glycated hemoglobin (HbA_1c_) concentration is an indicator of average blood glucose concentration over three months and has been suggested as a diagnostic or screening tool for diabetes [14]. It was observed that buspirone improved the glycosylated hemoglobin and glucose levels in STZ diabetic control rats. Thus, reduced glycosylated hemoglobin by buspirone improvement in glucose levels after glucose load indicate better efficacy of buspirone in glycemic control in type 1 diabetes.

Increased production of triglyceride-rich proteins and the diminished clearance by lipoprotein lipase result in hypertriglyceridemia, which is typically observed in diabetes [15]. In present study, buspirone treatment controlled the dyslipidaemia condition in STZ diabetic rats. Aripiprazole has been found to have intrinsic activity similar to buspirone and has partial 5HT1A agonist action both *in vivo* and *in vitro *[16]. Further, De Hert et al. [17] in a clinical trial reported that aripiprazole caused a significant decrease in total cholesterol levels, triglyceride levels, LDL-C, also non-HDL-C, Cholesterol/HDL-C, and LDL-C/HDL-C ratios. Thus, buspirone effect of decrease in lipid levels, being an agonist at 5-HT1A receptor like aripiprazole, is reasonable.

It is widely accepted that high levels of circulating cardiac damage markers like CK-MB and LDH represent a powerful and sensitive predictor of increased cardiac complications [18]. In present study, there was a significant increase in CK-MB and LDH levels and treatment with buspirone significantly decreased serum CK-MB and LDH levels. According to Kim et al. [19], increased dopamine concentration has been shown to be associated with cellular damage indicated by an elevated release of lactate dehydrogenase (LDH) from the cells. It has also been reported that buspirone blocks the dopaminergic autoreceptor which may increase dopamine concentration [20] and may result in increased LDH levels. Thus, the decrease in CK-MB and LDH levels by any of the unknown mechanisms of buspirone may prove to be beneficial in cardiac complications.

Higher levels of CRP predict cardiovascular events indicating a possible role for inflammation in the etiology of cardiovascular disease [21]. In the present study, buspirone treatment exhibited significant decrease in serum CRP levels. In a study of acute myocardial infarction patients, it was reported that insulin infusion attenuated the rise of CRP and enhanced fibrinolysis [22, 23]. Here, buspirone treatment in STZ diabetic rats showed increase in insulin levels, which might be the probable reason for decrease in serum CRP levels.

Several studies have shown that alterations in LV collagen gene level may be associated with cardiac fibrosis in diabetes [24, 25]. In present study, there was a significant increase in left ventricular collagen levels in STZ diabetic rats and treatment with buspirone significantly decreased left ventricular collagen levels in treated rats. It has been reported that 5-HT-enhanced production of type IV collagen by human mesangial cells is mediated by activation of PKC and subsequent increase in active TGF-beta activity and since buspirone is partial agonist of 5-HT, it modulates the effects of 5-HT and thus might have reduced collagen levels [26]. It has been reported that 5-HT-enhanced production of type IV collagen by human mesangial cells is mediated by activation of PKC and buspirone might have reduced collagen levels through its effect on 5-HT.

72% of the diabetic patients are found to have LVH, whereas only 32% of the nondiabetic patients show LVH [27]. In the present study, there was a significant increase in hypertrophic indices in STZ diabetic rats and treatment with buspirone reduced these hypertrophic indices. The results were also supported by the histopathological investigations which revealed a less reduction in extracellular space and less increase in cardiomyocyte diameter by buspirone treatment. Further, as reported earlier, buspirone decreases LV collagen level, which might be the plausible mechanism in controlling cardiac hypertrophy.

Bradycardia is frequently observed in STZ diabetic rats [28]. In the present study, buspirone significantly decreased blood pressure and increased heart rate in STZ diabetic treated rats. According to the reports by Goa and Ward [29], buspirone had no significant effect on BP and HR. However, conflicting reports by Hanson et al. [30], Taylor et al. [31], and Unrug et al. [32] suggest a decrease in mean arterial blood pressure and HR. Further, it has also been reported that chronic administration of buspirone in DOCA induced hypertensive rats significantly reduces BP and 5-HT1A agonists might possess antihypertensive action [33]. The effect of buspirone on CVS functions seems to be dependent on species, route, and dose [30].

The left ventricular dysfunction has been associated with decrease in rate or pressure development and decay [34, 35]. In present study, chronic treatment with buspirone significantly elevated decreased rate of pressure development and decay of STZ diabetic rats. Cardiac structural changes manifest as cardiovascular dysfunction. As mentioned earlier, buspirone prevents structural changes and cardiac hypertrophy and thus preserves rate of pressure development and decay and cardiac functioning.

STZ produces changes in renal function which may be due to the development of diabetes. In the present investigation, treatment with buspirone produced significant lowering of the elevated serum creatinine and urea levels in the STZ diabetic rats. Altered creatinine function impairs creatinine excretion in nephropathy [36]. Elevation of serum urea usually signifies decreased renal function. Hence, buspirone may be helpful in the delaying structural or functional changes in a diabetic kidney.

Oxidative stress stimulates cardiac damage, endothelial dysfunction, apoptosis, and collagen synthesis in the heart and contributes to the pathogenesis of myocardial remodeling and failure [37–39]. In the present study, buspirone treatment improved the oxidative stress parameters. Thampi et al. [40] have reported that 8-OH DPAT, a 5-HT1A receptor agonist, provided antioxidant protection, reduced ROS levels and raised MnSOD and GSH levels in both *in vivo* and *in vitro*. Thus, buspirone also being a 5-HT1A receptor agonist might be acting by the same mechanism as 8-OH DPAT and thus confers antioxidant protection in cardiac tissues.

## 5. Conclusions

In conclusion, our data suggests that buspirone prevents not only the STZ induced metabolic abnormalities but also cardiovascular complications as evident from the reduction in cholesterol, triglyceride, LDH, CK-MB, CRP, creatinine, urea and collagens levels, and decrease in cardiac hypertrophy and left ventricular hypertrophy which are the initial symptoms of congestive heart failure. The limitation of our study is that we have not evaluated the side-effects of this therapy. However, buspirone is beneficial wherein further studies could be carried out in primates in the future, which could serve as a potential antidiabetic agent in type 1 diabetes mellitus and also prevent its cardiac complications.

## Figures and Tables

**Table 1:** Effect of Buspirone on body weight, food and water intake, glucose levels and renal parameters.

Parameters	CON	COB	DIC	DIB
Body weight (gm)	217.5 ± 4.95	204.17 ± 4.36	209.37 ± 8.78*	220 ± 5.77^#^
Food intake (gm)	83.33 ± 4.21	78.33 ± 3.07	140 ± 12.11*	60 ± 9.31^#^
Water intake (mL)	116.67 ± 8.82	108.33 ± 10.77	386.67 ± 9.89*	91.67 ± 6.1^#^
Glucose (mg/dL)	75.08 ± 3.90	85.31 ± 4.85	317.14 ± 8.98*	210.25 ± 8.65^#^
Creatinine (mg/dL)	0.58 ± 0.03	0.6 ± 0.02	0.85 ± 0.06*	0.7 ± 0.02^#^
Urea (mg/dL)	45.85 ± 3.73	47.31 ± 3.07	65.61 ± 1.29*	51.22 ± 2.09^#^

*Significantly different from normal control (*P* < 0.05).

^#^Significantly different from diabetic control (*P* < 0.05).

Values are expressed as mean ± SEM of 6 experiments.

CON: normal control, COB: control treated with buspirone, DIC: diabetic control, and DIB: diabetic treated with buspirone.

**Table 2:** Effect of buspirone on lipid parameters.

Lipid parameters	CON	COB	DIC	DIB
Serum triglyceride (mg/dL)	43.03 ± 3.51	43.72 ± 5.83	193.85 ± 14.47*	134.73 ± 3.03
Total serum cholesterol (mg/dL)	71.32 ± 3.00	73.07 ± 4.85	93.63 ± 2.40*	83.57 ± 3.11
Serum VLDL-C (mg/dL)	8.89 ± 0.67	8.74 ± 1.17	38.77 ± 2.89*	26.95 ± 0.61
Serum LDL-C (mg/dL)	24.69 ± 1.36	26.79 ± 3.79	38.45 ± 1.92*	29.53 ± 0.83
Serum HDL-C (mg/dL)	29.79 ± 3.07	27.02 ± 2.00	13.21 ± 1.26*	18.62 ± 2.33
Log TG/HDL ratio	0.0069 ± 0.0006	0.0084 ± 0.0008	0.02806 ± 0.0023	0.0188 ± 0.0014

*Significantly different from normal control (*P* < 0.05).

^#^Significantly different from diabetic control (*P* < 0.05).

Values are expressed as mean ± SEM of 6 experiments.

CON: normal control, COB: control treated with buspirone, DIC: diabetic control, and DIB: diabetic treated with buspirone.

**Table 3:** Effect of buspirone on serum cardiac markers and hypertrophic parameter.

Parameters	CON	COB	DIC	DIB
Lactate dehydrogenase (U/L)	724.57 ± 61.32	750.82 ± 55.99	1044.17 ± 61.46*	822.35 ± 53.51^#^
C-reactive protein (mg/L)	0.99 ± 0.16	1.08 ± 0.17	2.78 ± 0.27*	1.79 ± 0.21^#^
Creatinine kinase-MB (U/L)	324.32 ± 16.17	358.12 ± 13.83	459.58 ± 12.58*	403.32 ± 18.77^#^
Cardiac hypertrophy index (mg/mm)	20.08 ± 0.17	20.16 ± 0.16	20.88 ± 0.13*	20.50 ± 0.08^#^

*Significantly different from normal control (*P* < 0.05).

^#^Significantly different from diabetic control (*P* < 0.05).

Values are expressed as mean ± SEM of 6 experiments.

CON: normal control, COB: control treated with buspirone, DIC: diabetic control, and DIB: diabetic treated with buspirone.

**Table 4:** Effect of buspirone on hemodynamic and cardiac oxidative stress parameters.

Parameters	CON	COB	DIC	DIB
Blood pressure (mm Hg)	1311 ± 4.07	134 ± 3.58	151 ± 4.83*	141 ± 2.36
Heart rate (beats/min)	381 ± 1010	392 ± 8.69	298 ± 12.84*	330 ± 9.39
Malondialdehyde (nmoles/mg protein)	0.66 ± 0.04	0.71 ± 0.06	1.67 ± 0.11*	1.20 ± 0.09^#^
Glutathione (*μ*g/mg protein)	2.17 ± 0.09	1.54 ± 0.05	0.97 ± 0.01*	1.47 ± 0.02^#^
Superoxide dismutase (U/mg protein)	4.11 ± 0.05	4.03 ± 0.04	2.53 ± 0.01*	2.77 ± 0.02^#^

*Significantly different from normal control (*P* < 0.05).

^#^Significantly different from diabetic control (*P* < 0.05).

Values are expressed as mean ± SEM of 6 experiments.

CON: normal control, COB: control treated with buspirone, DIC: diabetic control, and DIB: diabetic treated with buspirone.
